# Propofol inhibits the expression of Abelson nonreceptor tyrosine kinase without affecting learning or memory function in neonatal rats

**DOI:** 10.1002/brb3.1810

**Published:** 2020-09-01

**Authors:** Long Feng, Zhi‐Gao Sun, Qiang‐wei Liu, Tao Ma, Zhi‐Peng Xu, Ze‐Guo Feng, Wei‐xiu Yuan, Hong Zhang, Long‐he Xu

**Affiliations:** ^1^ Anesthesia and Operation Center Chinese PLA Medical School Beijing China; ^2^ PLA general hospital of Hainan Hospital Hainan China; ^3^ Department of Anesthesiology PLA Rocket Force Characteristic Medical Center Beijing China

**Keywords:** abelson nonreceptor tyrosine kinase, apoptosis, neonatal rat, neurocognitive dysfunction, propofol, ROS

## Abstract

**Objective:**

Propofol is one of the most commonly used intravenous drugs to induce and maintain general anesthesia. In vivo and in vitro studies have shown that propofol can affect neuronal growth, leading to apoptosis and impairing cognitive function. The Abelson nonreceptor tyrosine kinase (c‐Abl) is associated with both neuritic plaques and neurofibrillary tangles in the brains of patients with Alzheimer's disease and other neurodegenerative diseases. This study aimed to explore the effect of propofol on apoptosis and neurocognition through its regulation of c‐Abl expression in vivo and in vitro.

**Materials and Methods:**

In this study, primary hippocampal neurons were cultured and exposed to propofol at different concentrations. Protein expression was measured by Western blotting and coimmunoprecipitation. The c‐Abl transcription level was verified by fluorescence quantitative PCR. Reactive oxygen species (ROS) levels were detected by flow cytometry. In addition, an animal experiment was conducted to assess neuronal apoptosis by immunofluorescence staining for caspase‐3 and to evaluate behavioral changes by the Morris water maze (MWM) test.

**Results:**

The in vitro experiment showed that propofol significantly decreased c‐Abl expression and ROS levels. In addition, propofol has no cytotoxic effect and does not affect cell activity. Moreover, in the animal experiment, intraperitoneal injection of 50 mg/kg propofol for 5 days obviously decreased the expression of c‐Abl in the neonatal rat brain (*p* < .05) but did not significantly increase the number of caspase‐3‐positive cells. Propofol treatment did not significantly reduce the number of platform crossings (*p* > .05) or prolong the escape latency of neonatal rats (*p* > .05) in the MWM test.

**Conclusions:**

The present data suggest that reduced expression of this nonreceptor tyrosine kinase through consecutive daily administration of propofol did not impair learning or memory function in neonatal rats.

## Graphical abstract

Propofol multiple anesthesia may affect the learning and memory abilities of infants and young children. c‐Abl plays an important role in neurodegenerative diseases. Propofol multiple anesthesia has no effect on learning and memory function by inhibiting the expression of c‐Abl.

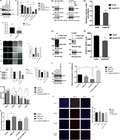

## 1 INTRODUCTION

Every year, millions of human newborns, infants, toddlers, and preschool‐aged children are exposed to anesthetic drugs due to the need for essential surgical or medical procedures (Reitman & Flood, 2011). Substantial evidence has indicated that general anesthetics can induce developmental neurotoxicity, including acute widespread neuronal cell death, followed by long‐term memory and learning abnormalities (Aksenov, Miller, Dixon, & Drobyshevsky, 2020; Bosnjak, Logan, Liu, & Bai, 2016; McInnis et al., 2002; Wang, Kaufmann, Sanchez‐Ross, & Johnson, 2000; Wang, Zhang, Liu, Paule, & Slikker, 2010). Propofol is one of the most commonly used intravenous drugs for the induction and maintenance of anesthesia and procedural and critical care sedation in children (Bosnjak et al., 2016). An increasing number of findings in animal models and cell culture studies have demonstrated that propofol can induce developmental neurotoxicity, raising serious concerns regarding the safety of pediatric propofol anesthesia (McInnis et al., 2002; Wang et al., 2000, 2010). The extent of this brain damage depends on the duration of anesthesia, the frequency of anesthesia exposure, and other factors related to the anesthesia protocol (Aksenov et al., 2020). Unfortunately, although many studies have found that propofol induces neurotoxicity in the developing animal brain, the specific mechanism of action remains largely unknown (Aksenov et al., 2020; Bosnjak et al., 2016; McInnis et al., 2002; Reitman & Flood, 2011; Wang et al., 2000, 2010).

Abelson nonreceptor tyrosine kinase (c‐Abl) was first discovered in Abelson murine leukemia virus by Ozanne, Wheeler, Zack, Smith, and Dale, (1982). The c‐Abl family tyrosine kinases include c‐Abl (Abl, Abl1) and Abl‐related genes (ARG, Abl2), which play a vital role in the actin cytoskeleton, the cell cycle, stress‐induced apoptosis, and cell cycle arrest (Hantschel & Superti‐Furga, 2004). Under normal conditions, c‐Abl is usually in an inactive state. Overexpression of c‐Abl has been shown to lead to apoptosis and changes in the cell cycle (Hantschel & Superti‐Furga, 2004; Sauravet al., 2016; Jones, Lu, & Lu, 2004). c‐Abl also plays important roles in neurodevelopment and neurodegeneration‐related diseases, and selective inhibition of c‐Abl expression had neuroprotective effects and improved cognitive behaviors in vitro and in vivo (Saurav et al., 2016; Guo, Kozlov, Lavin, Person, & Paull, 2010; Jones et al., 2004; Martin, Liu, Pipino, Chestnut, & Landek, 2009; Zukerberg et al., 2000). Schlatterer, Tremblay, Acker, and Davies (2011) found that overexpression of c‐Abl in neurons led to progressive neuronal loss and inflammation, which are observed in Alzheimer's disease (AD). In addition, inhibition of c‐Abl prevented both Purkinje cell death in Niemann–Pick C mice and Aβ42‐induced death in *Drosophila* neuronal cell cultures and led to a decrease in the level of p‐tau in Aβ‐PPsw/PSEN1ΔE9 mice (Cancino et al., 2011; Lin, Lin, & Juang, 2007). However, few studies have addressed whether anesthetics participate in postanesthesia learning and memory dysfunction by regulating the expression of c‐Abl.

Based on the above findings, we hypothesized that propofol exerts neuroprotective effects by inhibiting the expression of c‐Abl and regulates learning and memory function. To test this hypothesis, we used immunoblotting, immunoprecipitation, immunostaining, quantitative real‐time PCR, and flow cytometry to evaluate the effects of propofol on the expression of c‐Abl and the Morris water maze (MWM) test to evaluate neurocognitive function. The results obtained in this study may provide new insight into the mechanism of learning and memory dysfunction after anesthesia in surgery.

## 2 METHODS

### 2.1 In vitro study

#### 2.1.1 Primary hippocampal neuron culture

According to previous methods, primary hippocampal neurons were cultured in standard neurobasal/B27 medium (Beaudoin et al., 2012; Kaech & Banker, 2006). In brief, primary neuronal culture was prepared from the hippocampus of postnatal Sprague Dawley rats within 24 hr of birth (the rats were provided by Beijing Vital River Laboratory Animal Technology Co., Ltd.). The culture dishes were coated with poly‐D‐lysine (0.01 mg/ml, Sigma) 1 day before culture. In a laminar flow hood, scissors were used to harvest the mouse brains and hippocampi, which were placed in a dish containing Ca2+/Mg2+‐free Hank's balanced salt solution (HBSS) at 4°C. The tissue was resuspended in 4.5 ml of fresh dissection medium, and then, 0.5 ml of 2.5% trypsin solution was added. The solution was incubated in a cell incubator for 15 min and then placed in a dish containing fresh DMEM supplemented with fetal bovine serum (FBS, Gibco, USA). Four to six hours after plating, cells were examined under a microscope to ensure that most of the cells had settled on the substrate. The medium was replaced with fresh neurobasal/B27 medium (neurobasal medium (Gibco, USA) + 2% B27 medium (Gibco, USA) + 0.2 mM GlutaMAX‐I supplement (Gibco, USA) + 100 U/ml penicillin/streptomycin). One‐third of the medium was replaced every 3 days. Three days after plating, cytosine arabinoside (AraC) was added to the culture to a final concentration of 5 μM to inhibit glial proliferation. Cells could be used for experiments 7 days after plating.

In this study, cells at a density of 5 × 10^5^ cells per 60‐mm culture dish were used for Western blotting. The cell density for immunostaining was approximately 1–3 × 10^4^ cells per 6‐ or 12‐well culture dish.

#### 2.1.2 Propofol exposure

Previous studies suggested that the clinically relevant concentration of propofol for in vitro studies ranges from approximately 5 to 100 μM (Feiner et al., 2005; Vutskits, Gascon, Tassonyi, & Kiss, 2005).** **Thus, 50 and 100 μM (AstraZeneca UK Limited, NJ126) were used as clinically relevant concentrations of propofol in this study. A stock solution of 10,000 μM propofol was prepared. Cells in the propofol group were exposed to 50 or 100 μM propofol for 24 hr, while cells in the control group were treated with an equal volume of DMSO and/or 10 μM AMN‐107 (Novartis). Nilotinib (AMN‐107, Tasigna), a second‐generation c‐Abl tyrosine kinase inhibitor approved by the FDA in 2007 and used for chronic myeloid leukemia at daily doses of 300 mg to 1,000 mg, was used in this study (Weisberg et al., 2019). Because our laboratory often uses 10 μm AMN‐107 to treat cells for 21 hr, this concentration was also selected in this study.

#### 2.1.3 Plasmid construction and transfection

Nerve cells were cultured in a six‐well plate and could be transfected when they grew to approximately 70% density. First, half of the medium was aspirated and allowed to stand for 30 min, and then, the transfection reagents Optim + Lip2000 and Optim + Plastim were prepared. The entire process was performed on ice. After 5 min, the two reagents were evenly mixed. After 15 min, the reagents were added to a Petri dish and evenly mixed. After 6 hr, the remaining half of the transfected cells were placed in medium. Cells were collected 36–48 hr after the reagents were added. The Myc‐CRK plasmid and Flag‐c‐Abl plasmid were prepared by cloning into the pCMV‐Myc vector (Clontech). Neurons were transfected with the Myc‐CRK and/or Flag‐c‐Abl plasmid with Lipofectamine 3,000 according to the manufacturer's protocol (Life Technologies, Inc.). Neurons were digested and lysed after drug treatment, and the expression level of c‐Abl was determined in each group. The remaining lysates were coprecipitated and used to detect pTyr expression levels. The level of CRK phosphorylation (pTyr expression level) represents c‐Abl activity.

#### 2.1.4 Immunoprecipitation and immunoblotting

Cells for immunoblotting were lysed in buffer containing 50 mM HEPES (pH 7.4), 150 mM NaCl, 1% Nonidet P‐40, 0.1% deoxycholate, 0.05% SDS, 1 mM EGTA, and a protease inhibitor. The protein concentration was determined using a Bio‐Rad protein assay kit (Bio‐Rad, Hercules, CA, USA). Proteins were separated on an 8%–12% polyacrylamide gel and transferred to a methanol‐activated PVDF membrane (Bio‐Rad, Trans‐Blot *SD*, Semi‐Dry Transfer Cell, USA). Proteins were blocked for 1 hr in Tris‐buffered saline and Tween‐20 (TBST) containing 5% milk and subsequently probed with primary antibodies overnight at 4°C. After incubation for 1 hr with goat anti‐rabbit or goat anti‐mouse horseradish peroxidase (HRP)‐conjugated secondary antibodies, the antigen–antibody complexes were visualized by chemiluminescence (PerkinElmer Life Sciences). The following antibodies were used for immunoblot analysis: anti‐c‐Abl (Santa Cruz, SC‐131), anti‐β‐actin (Santa Cruz, SC‐1616), anti‐pTyr (4G10; Millipore, 16–105), anti‐c‐Abl (Santa Cruz, SC‐131), anti‐Myc (Santa Cruz, SC‐40), and anti‐Flag horseradish peroxidase (Sigma, A8592), anti‐Bcl2 (Abcam) and anti‐Bax (Abcam).

#### 2.1.5 Cellular RNA extraction and analysis by quantitative real‐time PCR

Total RNA was extracted from each group according to the instructions of the Qiagen RNeasy MiniKit. During the procedure, the operator must wear a laboratory coat, gloves, mask, and hat. At the same time, the pipette tip, centrifuge tube, and pipette used must be free of RNase contamination. The specific steps are as follows: Step 1: Collect an appropriate amount of cells, wash them with 1 × PBS, add 350 μl of RLT cell lysate, and purge the cells to lyse them. Then, add 350 μl of 70% ethanol to the lysate to precipitate the nucleic acid. Then, transfer the solution to an RNA adsorption column. Step 2: Place the RNA adsorption column into a centrifuge for centrifugation at >8,000 *g* for 30 s and then discard the liquid in the tube. Next, add 700 μl of RW1 to the adsorption column, centrifuge the column at >8,000 *g* for 30 s, and discard the liquid in the tube. Then, add 500 μl of RPE to the adsorption column, centrifuge the column at >8,000 *g* for 30 s, discard the liquid in the tube, repeat the above operation once, and then centrifuge the empty tube at >8,000 *g* for 5 min. Step 3: Place the adsorption column in a new RNase‐free 1.5 ml EP tube, add 30 μl of RNase‐free water to the center hole of the adsorption column, centrifuge the column at maximum speed for 2 min to elute the RNA, and then use spectrometry photometric detection to calculate the concentration of RNA in the sample.

Then, the extracted RNA was reverse‐transcribed into cDNA following the instructions of Promega's GoScriptTM Reverse Transcription System kit. The operation steps are as follows: First, add a solution containing 1 μg of RNA to a PCR tube, add 1 μl (0.5 μg) of each primer, including Oligo (dT) and Random Primer, use RNase‐free deionized water to achieve a final volume of 5 μL, and place the solution into a PCR machine at 70°C. After incubation for 5 min, quickly place the solution on ice to cool for 5 min and then centrifuge the solution in a microcentrifuge for 10 s, followed by placement on ice. Second, set up 15 μl of reverse transcription reaction mixture on ice. The reverse transcription reaction mixture system contains 5 × Reaction Buffer 4.0 μl, MgCl_2_ 4.5 μl, PCR Nucleotide Mix 1.0 μl, RNase inhibitor 0.5 μl, reverse transcriptase 1.0 μl, and deionized water 4 μl. Finally, mix the solution generated in the previous two steps and place it on the PCR instrument for 5 min at 25°C, 60 min at 42°C, and 15 min at 70°C. The average values from three independent experiments with standard deviations (SDs) (error bars) were calculated, and *p* values were determined by ANOVA.

#### 2.1.6 Flow cytometry analysis

After treating nerve cells with different drugs, the cells were fully digested with trypsin and then washed twice with 4°C PBS solution. Next, 300 ml of PBS solution containing 5% fetal bovine serum to resuspended the cells, and 5 ml of the solution prepared with PBS was mixed drop by drop with 70% ethanol, followed by storage at −20°C overnight. Then, the cells were washed twice with PBS, and the excess liquid was completely removed under a vacuum pump. The cells were transferred to an EP tube, and 300 μl of RNase solution was added. After 30 min, the neurons were washed once with PBS, resuspended in 3% FBS, and then tested on the machine. The fluorescence intensity of the cells was assayed using ModFit LT software.

#### 2.1.7 Analysis of cellular reactive oxygen species (ROS) levels

The Reactive Oxygen Species Assay Kit (Reactive Oxygen Species Assay Kit) (Beyotime, NO, 011520200402) uses the fluorescent probe DCFH‐DA for active oxygen detection. DCHF‐DA does not have fluorescence, but DCFH‐DA can be hydrolyzed and split by lipase to form DCFH after penetrating the cell membrane and entering the cell; then, the fluorescence of DCFH is detected to determine the strength of the oxidative stress response. In this experiment, the cultured primary hippocampal neuron cells were cultured in a six‐well plate, and then, the cultured neurons were treated with different drugs for 24 hr, washed once with PBS, fully digested with trypsin, and collected. Then, DCFH‐DA was diluted with serum‐free medium according to the instructions at a ratio of 1:1,000, and the final drug concentration was 10 μM/L. Primary neurons were plated in 30‐mm culture dishes and stained in situ according to the instructions of a DCFH‐DA ROS assay kit (Beyotime Biotechnology) (10 μM, 37°C, 20 min in the dark). To ensure that the test solution fully contacted the cells, the solution was gently mixed every 3–5 min. Cells were washed twice with serum‐free DMEM, trypsinized, and centrifuged at 1,000 *g* for 1 min at 25°C to obtain cell pellets. Finally, with a Guava easyCyte 6TH flow cytometer, 5,000–10,000 cells per sample were detected in three independent experiments, and the average fluorescence intensity of the cells in the GRN‐HLog channel was analyzed. A 488‐nm excitation wavelength and 525‐nm emission wavelength were used on the flow cytometer.

#### 2.1.8 Cell viability assays

The 3‐(4,5‐dimethylthiazol‐2‐yl)‐5‐(3‐carboxymethoxyphenyl)‐2‐(4‐sulfophenyl)‐2H‐tetrazolium (MTS) assay is one of the commonly used colorimetric absorption assays based on the ability of dehydrogenase from viable cells to produce the brown soluble formazan detectable at 490 nm(20). This study used the Cell Titer96® AQueous One Solution Cell Proliferation Assay (Promega). Before the experiment, the main equipment, including 96‐well plates, a rifle gun, a microplate reader, and so on, was prepared. The specific experimental procedure was as follows. First, the cultured primary hippocampal neuron cells were plated in 96‐well plates. After the cells were plated and grown for 1 week, different drug treatments were added at the same time point, and each treatment group had three multiwells. After 24 hr of drug treatment, the cultured cells were removed from the incubator and washed twice with warm PBS. Since the MTS staining solution must be stored in the dark at −20°C, the staining solution should be thawed in warm water before testing cell viability. Then, the optical absorbance of the samples was measured by MULTISCAN GO (Thermo Scientific) with a background control as the blank according to the user manual.

### 2.2 Animal study

#### 2.2.1 Animals

All experiments were performed in accordance with approved institutional animal care guidelines from the Chinese People's Liberation Army General Hospital. All neonatal rats were ordered from Speyford (Beijing) Biotechnology Co., Ltd., and laboratory experiments began 2 days after the rats were received. The rats were housed in a temperature‐controlled (22–23°C) room under a 12‐hr:12‐hr light:dark cycle (lights on at 8:00 a.m.) with free access to food and water.

We used postnatal day 7 (PND7) male neonatal rats for all experiments because at this time point, brain development is at its peak, and the rats are vulnerable to anesthesia‐induced apoptotic damage (Li, Mu, & Gage, 2009). Before the study, all PND7 male neonatal rats were randomly assigned to the control group, AMN‐107 group, propofol group, or propofol + AMN‐107 group, each of which contained 12 rats. The minimum number of rats in each group was determined by power analysis. The animal sample size for this experiment was mainly determined based on the research of Peng et al. (2014). PND7 rats in the treatment groups were given 50 mg/kg propofol (AstraZeneca UK Limited, NJ126) by intraperitoneal (IP) injection five time at 24‐hr intervals (50 mg/kg/injection × 5 injections). At the same times, rats in the control group received an equal volume of DMSO. Rats in the AMN‐107 and propofol + AMN‐107 groups were given 5 mg/kg AMN‐107 by IP injection 21 times at 24‐hr intervals (5 mg/kg/injection × 21 injections). Temperature probes were used to control the temperature at 37°C using integrated heating plates under the chambers. The neonatal rats were given oxygen to prevent hypoxia.

The animals were sacrificed after IP injection of propofol on five consecutive days. Before immunofluorescence staining, the neonatal rats were sacrificed by dislocation and fixed with paraformaldehyde, and the whole brain was taken for subsequent use. In addition, suckling rats used for Western blot analysis were anesthetized by an IP injection of chloral hydrate and then decapitated, and brain tissue was removed. The bilateral hippocampus was dissected and frozen in a −80°C refrigerator for later use. The MWM test was conducted with the remaining rats after 30 days of propofol treatment.

#### 2.2.2 Anesthesia

The dose of propofol used was determined based on the results of previous studies (Cattano, Young, Straiko, & Olney, 2008; Lanigan, Sury, Bingham, Howard, & Mackersie, 1992) reporting that a minimal effective dose of ≥50 mg/kg triggered a significant neuroapoptosis response. Therefore, propofol was administered at 50 mg/kg for 5 consecutive days in this study. Nilotinib (AMN‐107, Tasigna), a second‐generation c‐Abl tyrosine kinase inhibitor approved by the FDA in 2007 and used for chronic myeloid leukemia at daily doses of 300 mg to 1,000 mg, was used in this study (Weisberg et al., 2019). Administration of this drug at doses of 5–10 mg/kg by daily IP injection for up to 3 consecutive weeks was effective in models of neurodegeneration (Fowler et al., 2019).

#### 2.2.3 MWM test

Hippocampal‐dependent spatial memory ability was tested using the MWM test. A different group of rats was tested at 1 month after birth. A circular pool (180 cm diameter, 50 cm depth) painted black was filled with water to a depth of 30 cm. The water temperature was maintained at 24 ± 1°C. An invisible platform (10 cm diameter) was submerged 1 cm below the water surface and placed in the center of area VII, which refers to six starting positions marked at equal distances around the edge of the pool labeled I, II, III, IV, V, and VI. The experiments were conducted over four consecutive days in a dark, quiet laboratory. All rats (six per group) were trained six times per day, and random starting positions were used for each rat. When a rat found the platform, it was allowed to stay on the platform for 3 s. If a rat did not find the platform within 60 s, the rat was gently guided to the platform and allowed to stay on the platform for 3 s, and the latency time to find the hidden platform was recorded as 60 s. On the fifth day, the hidden platform was removed, and the rat was placed in the opposite quadrant. Rats were allowed to swim freely for 60 s. The number of times that the rat swam across the previous platform area and the time that the rat stayed in the target quadrant up to 60 s was recorded. Each animal's path was tracked with a computerized video system. After every trial, each rat was placed on a heating plate for 1–2 min until it was warm before it was returned to its chamber.

#### 2.2.4 Statistical analysis

At least three independent experiments were carried out to generate each data set, and statistical significance in each case was calculated using one‐way ANOVA as indicated in the figure legends. Tukey's post hoc test was used for comparisons between groups. Students–Newman–Keuls and least significant difference statistical methods were used for pairwise comparisons among multifactor groups. Differences with *p* < .05 and *p* < .01 were considered statistically significant.

## 3 RESULTS

### 3.1 Propofol reduced c‐Abl protein expression and transcription levels

**Figure 1:**
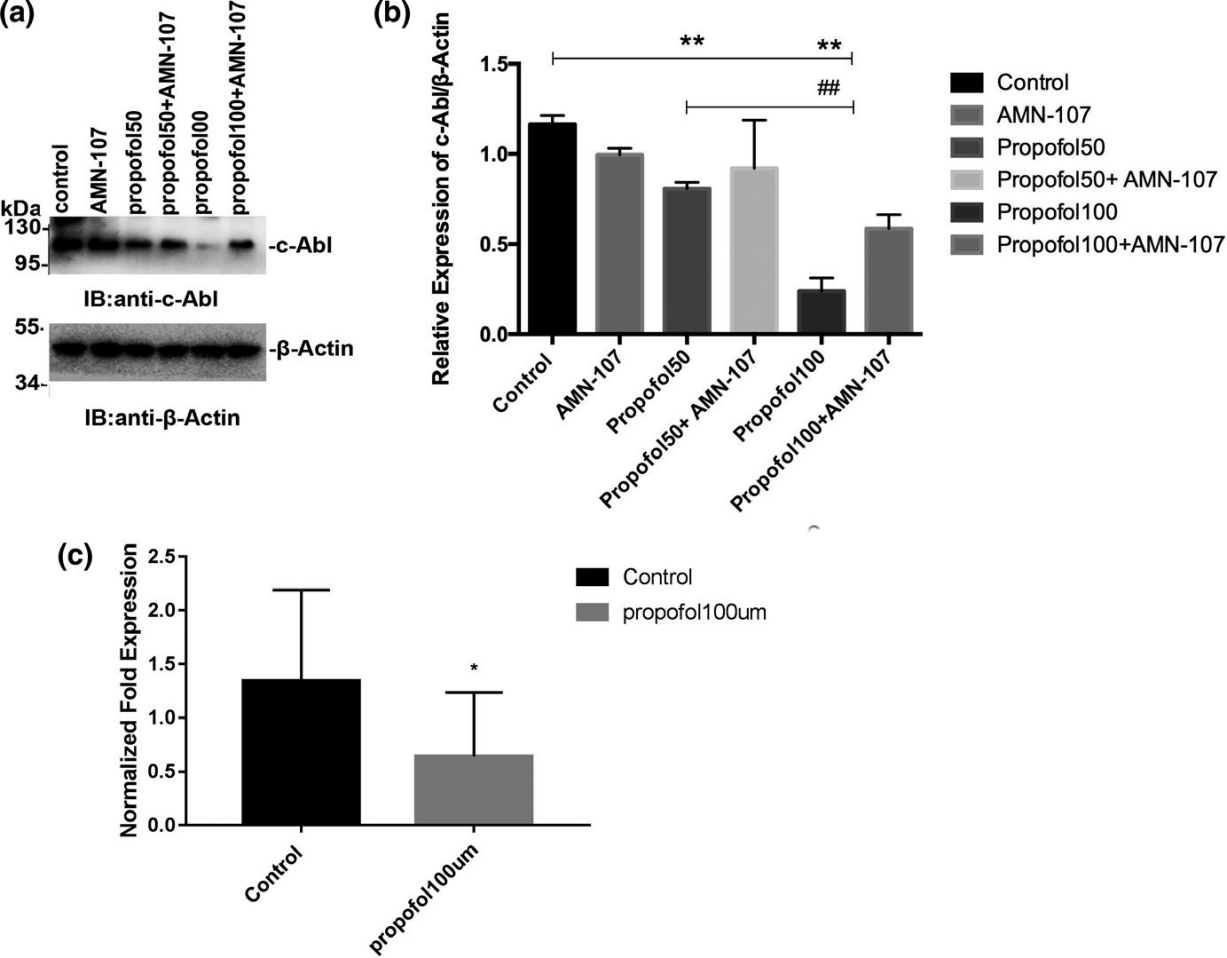
Propofol reduced c‐Abl protein expression and transcription levels. (a) Immunoblotting to detect the expression of target protein c‐Abl. (b) The relative expression level of c‐Abl. (c) The level of mRNA transcription of c‐Abl in nerve cells changed after drug treatment. All data are presented as the $\bar{x}$ ± s (*p < .05 vs. the control group; **p < .01 vs. the control group;^##^ *p* < .01 vs. the propofol group). All quantitative data are represented as the Mean ± *SD* of three independent experiments

To analyze whether propofol can regulate the expression of c‐Abl, neurons were exposed to propofol at different concentrations for 24 hr. Exposure to 50 μM and 100 μM propofol significantly decreased the expression of c‐Abl (*p* < .01) (Figure 1a,b). In addition to its effect on the c‐Abl protein level, we investigated the effect of propofol on the c‐Abl transcription level in neurons by rt‐PCR. Propofol significantly reduced transcription of the kinase c‐Abl in neurons (*p* < .05) (Figure 1c). All quantitative data are represented as the mean ± *SD* of three independent experiments.

### 3.2 Propofol enhanced c‐Abl activity, significantly increased the ubiquitination level, and reduced ROS levels

**Figure 2:**
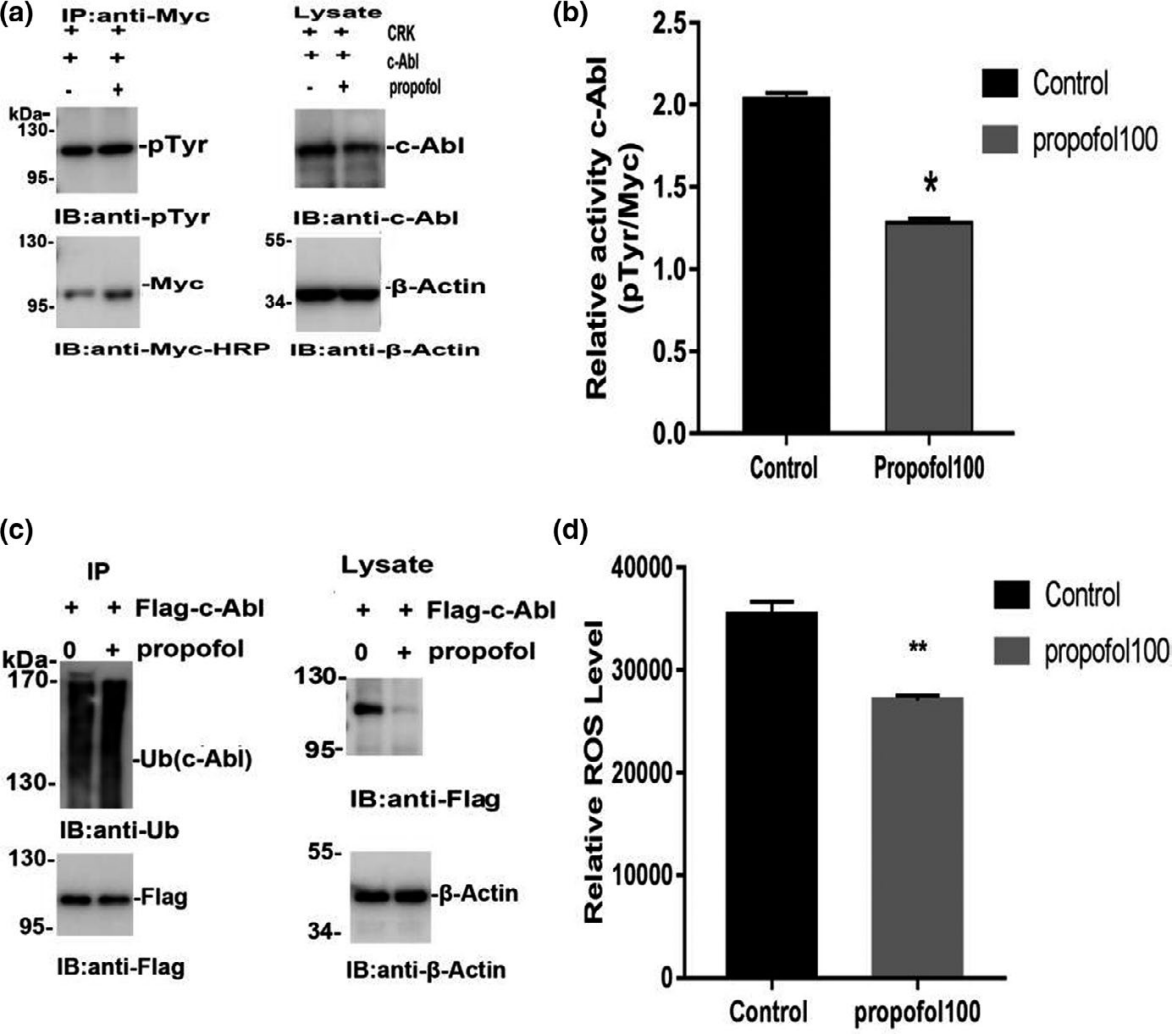
Propofol enhances c‐Abl activity, significantly increases its ubiquitination level, and decreases the ROS level. (a) Target protein c‐Abl, Myc, and pTyr bands. (b) The relative activity of c‐Abl. (c) Ub and c‐Abl expression bands, and the base standard β‐Actin expression bands. (d) Neuronal ROS levels changed after drug treatment. All data are presented as the $\bar{x}$ ± s (*p < .05 vs. the control group; **p < .01 vs. the control group). All quantitative data are represented as the Mean ± *SD* of three independent experiments

The kinase c‐Abl can be activated by multiple stimuli, leading to cytoskeletal reorganization, which is required for cell morphogenesis, mobility, adhesion, and polarity. The activity of c‐Abl plays a critical role in the regulation of apoptosis and has been linked to the pathogenesis of neurodegenerative diseases. To study the activity of c‐Abl, we first constructed the Myc‐CRK plasmid. The plasmid was then transfected into neurons, which were treated with propofol for 24 hr, and then, the Myc‐CRK phosphorylation level (pTyr expression level) was detected by coimmunoprecipitation. Neurons were digested and lysed after drug treatment to determine the expression level of c‐Abl in each group. The remaining lysates were coprecipitated and used to detect pTyr expression levels. The phosphorylation level of CRK (pTyr expression level) represents c‐Abl activity. The results of the coimmunoprecipitation experiment showed that propofol significantly increased the activity of c‐Abl (*p* < .05) (Figure 2a,b). The ubiquitin–proteasome system is the main method by which the body degrades proteins. To further study how propofol treatment decreased the expression of c‐Abl, we also studied the ubiquitination level of c‐Abl after propofol treatment. We transfected neurons with exogenous Flag‐c‐Abl and measured the level of c‐Abl ubiquitination. Propofol treatment significantly increased the neuronal c‐Abl ubiquitination level (Figure 2c). In addition, the increase in c‐Abl expression was mainly due to an increase in ROS levels, which is also the main cause of anesthetic‐induced neurotoxicity. The intracellular ROS levels of neurons treated with propofol at the indicated concentration for 24 hr were assessed by DCFH‐DA fluorescence intensity using a flow cytometer. ROS levels were significantly lower in the groups treated with propofol for 24 hr than in the control group (*p* < .01) (Figure 2d). All quantitative data are represented as the mean ± *SD* of three independent experiments.

### 3.3 The effects of propofol on the apoptosis and activity of primary hippocampal neurons after 24 hr

**Figure 3:**
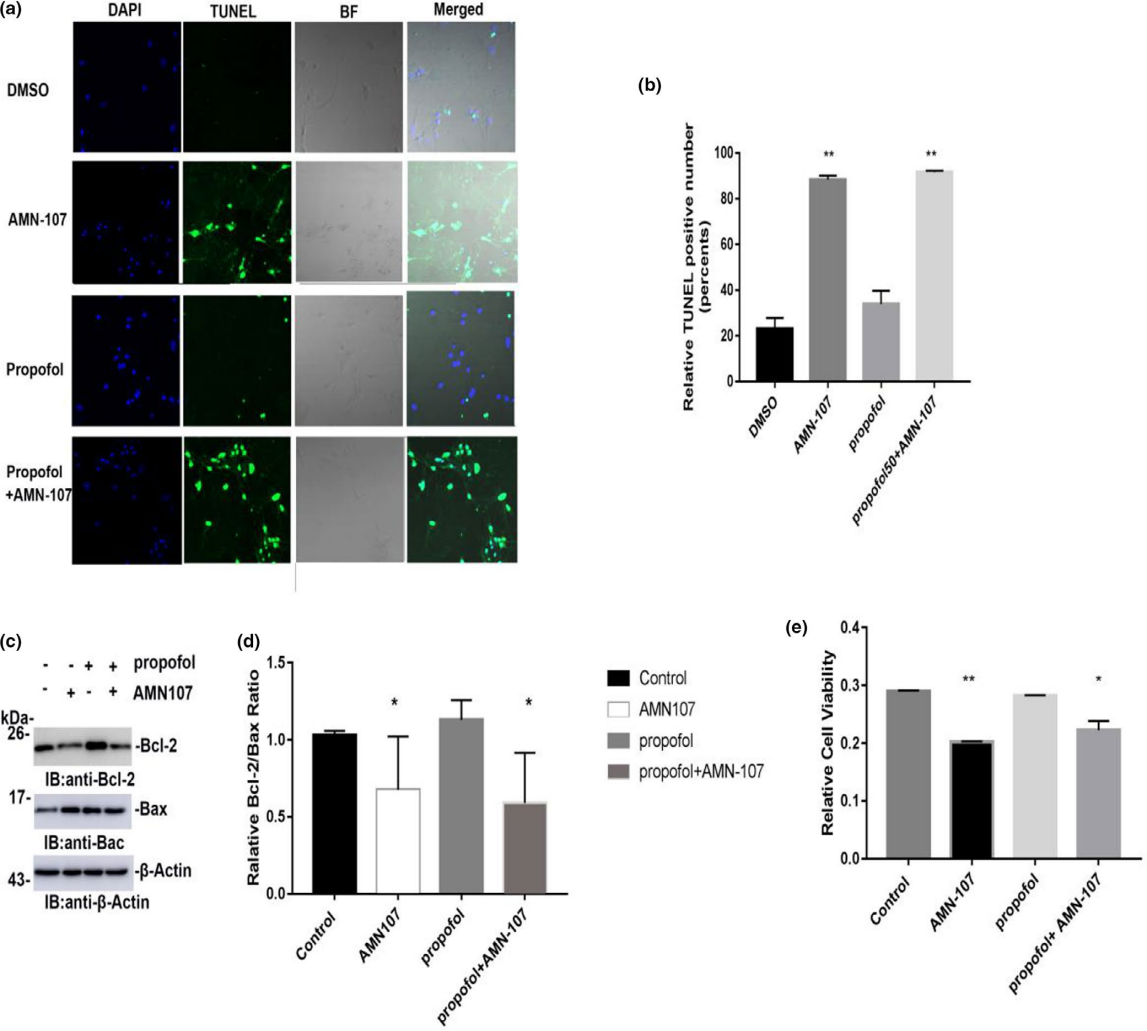
The effect of propofol on primary hippocampal neuronal cells after 24 hr on apoptosis and activity. (a) Immunofluorescence staining of TUNEL and DAPI of nerve cells in each group. The blue fluorescence is the nucleus of nerve cells stained by DAPI, and the green fluorescence shows TUNEL‐stained nerve cells. (b) The number of TUNEL staining‐positive cells in each group. (c) Bcl‐2 and Bax protein bands and β‐Actin bands of nerve cells. (d) Relative Bcl‐2/Bax ratio. (e) The relative activity changes of nerve cells in different drug treatment groups. The results are presented as $\bar{x}$ ± s (*n* = 3). *p < .05 versus the control group; **p < .01 versus the control group; One hundred neurons in each immunofluorescence staining were counted, after which the number of TUNEL‐positive cells was determined

The activity of c‐Abl plays a key role in the regulation of apoptosis. We examined the effect of propofol on apoptosis after inhibiting c‐Abl expression by immunofluorescence staining. DAPI and TUNEL staining was performed 24 hr after exposure to 100 μM propofol, and cell morphology and TUNEL positivity were observed under a confocal laser microscope. One hundred neurons in the immunofluorescence‐stained membranes were counted, and the number of TUNEL‐positive cells was determined. Compared with the control group, the positive number of TUNEL cells did not increase significantly after exposure to propofol for 24 hr (*p* > .05) (Figure 3a,b). The AMN‐107 group and propofol + AMN‐107 group had significantly increased numbers of TUNEL‐positive neuronal cells, and the difference compared with the control group was statistically significant (*p* < .01) (Figure 3a,b). In addition, we also tested the effect of 100 μM propofol on the Bcl‐2/Bax ratio by immunoblotting. The results showed no significant change in the Bcl‐2/Bax ratio of propofol‐treated primary hippocampal neurons after 24 hr and no significant difference compared with the control group (*p* > .05) (Figure 3c,d). However, the AMN‐107 group and propofol + AMN‐107 group had significantly reduced Bcl‐2/Bax ratios, with significant differences between these groups and the control group. The AMN‐107 group and propofol + AMN‐107 group showed significant neurotoxicity (*p* < .01) (Figure 3c,d). The neurotoxic effect caused by AMN‐107 may be related to the concentration and/or exposure time. In addition, MTS test results showed no significant change in neuronal activity after propofol treatment and no statistically significant difference compared with the control group (*p* >.05) (Figure 3e).

### 3.4 Hippocampal neurons from neonatal rats exposed to propofol exhibited decreased expression of c‐Abl, but neuronal apoptosis was not induced in the animals

**Figure 4:**
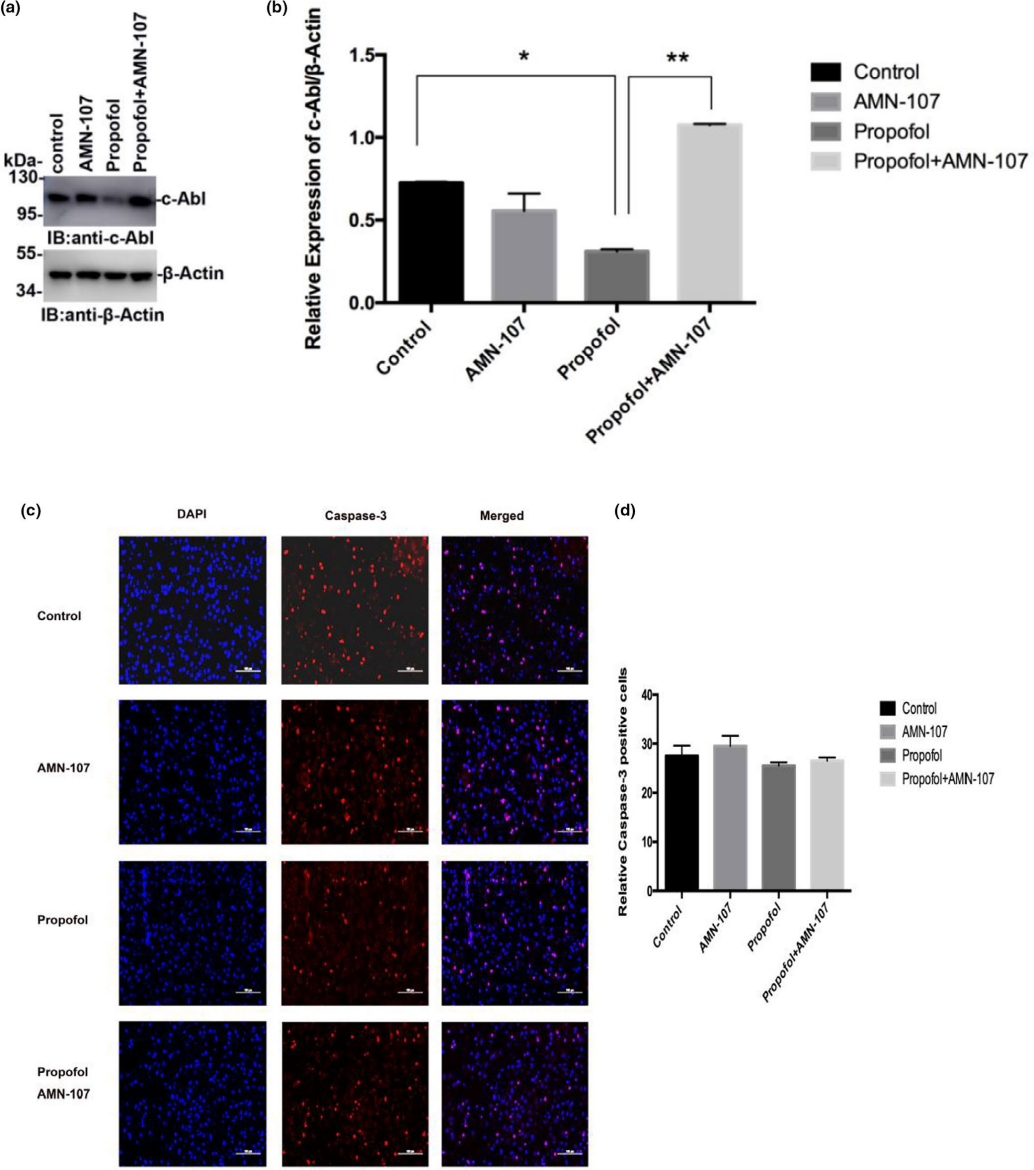
Propofol treatment significantly decreased c‐Abl expression but did not affect caspase‐3‐positive cell numbers in the hippocampus of neonatal rats. (a) Band indicating c‐Abl and β‐Actin expression in the hippocampus of neonatal rats. (b) Relative expression of c‐Abl in the hippocampus of neonatal rats. The results are presented as $\bar{x}$ ± s (*n* = 3). **p < .01 versus the control group; ^##^ *p* < .01 versus the propofol group. All quantitative data are represented as the mean ± *SD* of three independent experiments. (c) Immunofluorescence staining results of the hippocampus in the acute phase of different drug treatment groups after administration, in which blue is the nerve cell nucleus stained by DAPI and red is the positive nerve cell stained by caspase‐3. (d) The number of caspase‐3‐positive neurons stained between different groups. Scale bar: 100 µm. The results are presented as $\bar{x}$ ± s (*n* = 6). *p < .05 versus the control group. One hundred neurons in each immunofluorescence staining film were counted, after which the number of caspase‐3‐positive cells was determined

To further investigate whether propofol affects learning and memory function by regulating the expression of c‐Abl, PND7 neonatal rats were intraperitoneally injected with propofol for 5 consecutive days, and the hippocampus was removed during the acute phase after propofol administration and used to detect changes in c‐Abl expression by immunoblotting. The results in this experiment showed that the expression of c‐Abl was significantly decreased in the propofol‐treated group compared with the control group (*p* < .05) (Figure 4a,b). In addition, the expression of c‐Abl was significantly higher in the propofol + AMN‐107 group than in the propofol group (*p* < .01) (Figure 4a,b). Furthermore, to investigate the effects of propofol on apoptosis in the neonatal rat hippocampus, we analyzed caspase‐3 expression in the hippocampus of PND7 rats after exposure to propofol. One hundred neurons in each immunofluorescence‐stained film were counted, and the number of caspase‐3‐positive cells was determined. Immunofluorescence staining in the hippocampus of neonatal rats showed that the expression of caspase‐3 in the propofol‐treated group was not significantly increased compared with that in the control group (*p* > .05) (Figure 4c,d). These results suggested that the concentration of propofol used in this experiment (50 mg/kg) could not induce apoptosis by downregulating c‐Abl expression.

### 3.5 Effects of five consecutive days of propofol injection on learning and memory function of neonatal rats

**Figure 5:**
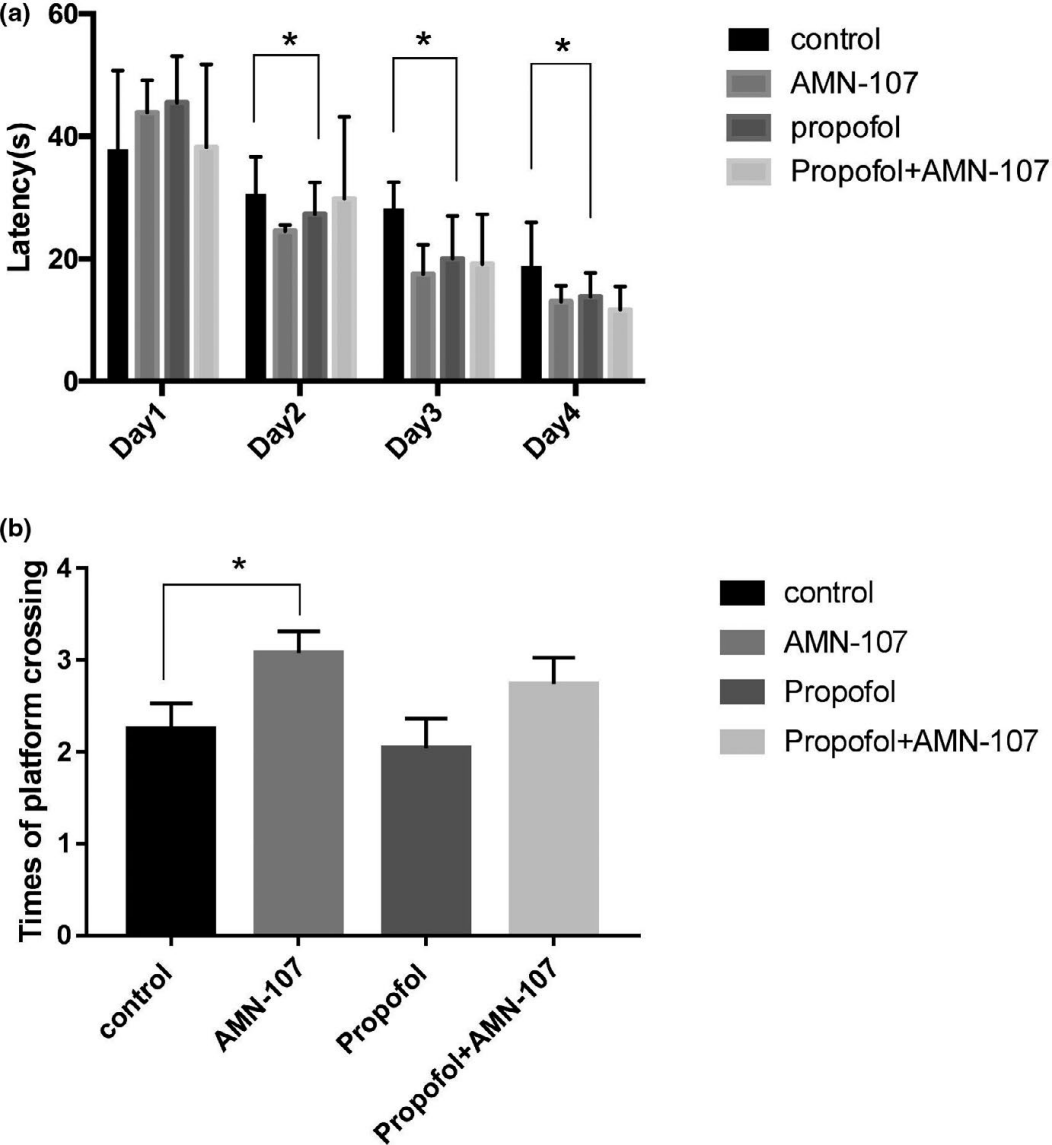
Effect of five consecutive days of propofol injection on learning and memory function of neonatal rats. (a) The escape latency of the water maze; (b) the number of times the water maze crosses the target quadrant. The results are presented as $\bar{x}$ ± s (*n* = 6). *p < .05 versus the control group

In addition to studying changes in c‐Abl expression, we studied the biological effects of downregulated c‐Abl expression after 5 consecutive days of IP propofol injection through the MWM test. Escape latency in the MWM test is a very important reference index and represents an animal's spatial learning and memory abilities. The escape latency during the four consecutive days of training in the MWM was analyzed by a continuous‐variable multivariate method, and the results suggested that the latency to locate the hidden platform in the propofol group was not longer than that in the other groups (*p* = .108, *p* > .05) (Figure 5a). However, comparison of the escape latency of the groups on the second, third, and fourth days of training showed that the escape latency of the propofol group was shorter than that of the control group at each time point, and the differences in escape latency between the groups were statistically significant (*p* < .05) (Figure 5a). Moreover, the more times that an animal traverses the original platform position within a certain period of time, the better its spatial learning and memory ability are. In memory retrieval tests, the number of crossings over the platform site within 60 s was not significantly reduced in the propofol group compared with the control group (*p* > .05) (Figure 5b). However, compared with the control group, the AMN‐107 group performed a significantly higher number of target quadrant platform crossings (*p* < .05) (Figure 5b).

## 4 DISCUSSION

The results of this study show that the intravenous anesthetic propofol can significantly reduce the expression of c‐Abl but increase the level of ubiquitination. In addition, propofol has no cytotoxic effect and does not affect cell activity. Recently, c‐Abl has been linked to oxidative stress‐induced neuronal cell death through Cdk5/GSK3β activation and Tau hyperphosphorylation and through p73 upregulation (Cancino et al., 2011). In addition, Xiao et al. (2011) proved that c‐Abl can be regulated by oxidative stress, leading to neuronal cell death. Therefore, we detected the ROS level of neurons after treatment with propofol, which was significantly lower than that in the control group, suggesting that the above changes may be related to a reduction in ROS levels by propofol. Furthermore, animal experiments showed that IP injection of propofol at a subclinical dose for five consecutive days significantly reduced the expression of c‐Abl in the hippocampus of neonatal rats without causing an increase the number of cells positive for caspase‐3. In addition, through the MWM test, we found that the above changes did not lead to impaired learning or memory in neonatal rats.

Propofol is well known for its neuroprotective effects, which are derived from its antioxidant properties (Bosnjak et al., 2016; Grasshoff & Gillessen, 2002; Qiao et al., 2017; Solaroglu et al., 2004; Zhou et al., 2016). Both a single propofol dose and repeated propofol dosing during brain development cause neuronal apoptosis and degeneration, which may be connected to ROS levels (McInnis et al., 2002; Satomoto et al., 2009; Yu, Jiang, Gao, Liu, & Chen, 2013). Our results demonstrate that propofol can downregulate the expression of c‐Abl by reducing ROS levels. In addition, propofol has no cytotoxic effect and does not affect cell activity. The results are consistent with those in the study by Palanisamy et al. (2016) and Yuan et al. (2016) in which treatment with propofol did not induce necrotic cell death or apoptosis. Prolongation of exposure to 24 hr only transiently impaired proliferation. Imam et al. (2011) found that oxidative and dopaminergic stress in primary neuronal cultures led to c‐Abl activation and expression, with subsequent tyrosine phosphorylation of parkin, leading to loss of the protective E3 ubiquitin ligase activity of parkin and accumulation of AIMP2 and FBP. However, most studies suggest that the expression level of c‐Abl is negatively correlated with the change in c‐Abl activity (Nagar et al., 2003, 2006; Pluk, Dorey, & Superti‐Furga, 2002; Thai et al., 2011). The regulation principle may be related to the quantity‐effect regulation mechanism. The above results also suggest that propofol prevents c‐Abl expression through its antioxidant activity (Bosnjak et al., 2016; Grasshoff & Gillessen, 2002; McInnis et al., 2002; Qiao et al., 2017; Satomoto et al., 2009; Solaroglu et al., 2004; Yu et al., 2013; Zhou et al., 2016). Moreover, animal experiments have confirmed that repeated propofol anesthesia can reduce the expression of c‐Abl without causing apoptosis. In vivo and in vitro studies have demonstrated that propofol plays an important role in the process of dependent cell death and can prevent calcium overload caused by mitochondrial depolarization by inhibiting excessive production of ROS (Yuan et al., 2016). The antioxidation mechanism of propofol can also reduce iron overload by regulating iron metabolism, inhibit oxidative stress and the inflammatory response, and avoid neuronal damage (Yuan et al., 2016). Zhao, Li, Wang, Zhao, and Wang (2016) found that ketamine alone significantly induced neuronal apoptosis and caused cognitive learning impairment in rats. However, ketamine combined with propofol can reduce the occurrence of apoptosis and improve cognitive decline. These results were mainly related to the antioxidant protective effect of propofol in the brain. Similar studies have also found that propofol increased ketamine‐induced cognitive dysfunction and suppressed immune function (Cao, Zhang, Ai, Zhang, & Li, 2014).

Oxidative stress activates and enhances the expression of c‐Abl. The c‐Abl family of tyrosine kinases includes c‐Abl (Abl, Abl1) and Abl‐related genes (ARG, Abl2), which play a vital role in the actin cytoskeleton, the cell cycle, stress‐induced apoptosis, and cell cycle arrest (Hantschel & Superti‐Furga, 2004). Under normal conditions, c‐Abl is in an inactive state. When overexpressed, c‐Abl can cause apoptosis and changes to the cell cycle. c‐Abl overexpression can be caused by various known contributors to neurodegenerative pathology, including oxidative stress, genotoxic stress, TNF‐α, Aβ fibrils, and neurofibrillary tangles (NFTs), and activation of c‐Abl by these factors can lead to apoptosis and cell cycle arrest (Schlatterer, Acker, and Davies, 2011; Schlatterer, Tremblay, et al., 2011). In addition, c‐Abl is important in neurodevelopment and neurodegeneration‐related diseases, and selective inhibition of c‐Abl expression has neuroprotective effects and improves cognitive behaviors (Saurav et al., 2016; Guo et al., 2010; Jones et al., 2004; Martin et al., 2009; Zukerberg et al., 2000). Our study demonstrated that propofol can inhibit the expression of c‐Abl without causing neuroapoptosis by reducing ROS levels. This finding is similar to the results of Schlatterer, Acker, et al., (2011) and Schlatterer, Tremblay, et al., (2011) who also found that overexpression of c‐Abl led to neuron loss. ROS can activate the kinase ataxia telangiectasia mutated (ATM) through oxidative stress, which further activates c‐Abl (Meltser, Ben‐Yehoyada, & Shaul, 2011). In addition, a subtype of protein kinase C can activate and phosphorylate c‐Abl under H_2_O_2_ stimulation (Guo et al., 2010; Sun, Wu, Datta, Kharbanda, & Kufe, 2000). Some authors have even suggested that ROS also directly activate c‐Abl (Brahmachari et al., 2017). Alvarez, Sandoval, Leal, Castro, and Kosik (2004) also found that the kinase c‐Abl could be activated under oxidative stress in a neuronal cell culture. In contrast, blockade of c‐Abl expression was reported to attenuate ROS production in neuron cells treated with a prion peptide fragment (Pan et al., 2014).

Three recent human studies, the General Anesthesia Spinal (GAS) study, Pediatric Anesthesia Neurodevelopment Assessment (PANDA) study, and Mayo Anesthesia Safety in Kids (MASK) study, found that exposure to general anesthesia for a short duration does not result in detectable neurodevelopmental impairment (Davidson et al., 2016; McCann et al., 2019; Warner et al., 2018). However, the risk of cognitive and behavioral impairment is especially high among children with repeated anesthetic exposure. Our results suggest that IP injection of propofol for five consecutive days can significantly reduce the expression of c‐Abl in the hippocampus without causing a neuroapoptotic response. Experiments by Sun, Yuan, Liu, Zhang, and Tu (2019) also indicated that 50 mg/kg propofol caused no neuronal structural changes in P7 rats and did not upregulate caspase‐3 expression. However, Cattano et al. (2008) found that a single IP injection of propofol at a dose of 50 mg/kg in 5‐ to 7‐day‐old neonatal rats caused significant cell death in the cortex and caudate. These findings also show that the minimal effective dose of propofol (subclinical dose, 50 mg/kg) required to cause a significant neuroapoptotic response is approximately one‐fourth of the dose required to induce a surgical plane of anesthesia in infant mice. These conflicting results may result from differences in the administration method, treatment interval, and part of the brain examined. The extent of brain damage depends on the duration of anesthesia, the frequency of anesthesia exposure, and other factors related to the anesthesia protocol (Aksenov et al., 2020). In addition, Amrock, Starner, Murphy, and Baxter (2015) found that general anesthetic‐induced neurotoxicity has a “threshold effect,” with a longer duration of anesthesia corresponding to a greater likelihood that the nerves in the brain will initiate repair processes and reduce damage.

This study indicated that repeated administration of propofol did not cause spatial memory impairment during critical periods of neurodevelopment. The results of several more recent studies have generally been consistent with these original findings. Treatment with propofol (50 mg/kg) at an early postnatal age was also found to have nonsignificant effects on neonatal neurodegeneration and subsequent neurodegeneration (Lee et al., 2008). Fredriksson, Pontén, Gordh, and Eriksson (2007) also found that propofol at 60 mg/kg induced neurodegeneration in the brains of 10‐day‐old mice. However, some studies have presented inconsistent results. Zhang, Liang, Sun, and Pei (2017) found that the use of a dose of 40 mg/kg and repeated administration of propofol (0–120–240 min) induced learning and memory impairment in PND7 rats. The difference in these results is mainly due to the use of different dosing regimens. Zhang and colleagues mainly simulated clinical continuous intravenous anesthesia with propofol for infants and young children. However, we mainly simulated repeated exposure to anesthesia, which may be necessary in clinical practice, such as in children with multiple congenital diseases or those undergoing plastic surgery who need repeated treatment and anesthesia. However, many studies have shown that propofol can induce apoptosis of neurons in the developing brains of different species (Cattano et al., 2008; Creeley et al., 2013; Pearn et al., 2012; Tu et al., 2011). Chen, Deng, Wang, and Liu (2016) found that single exposure to propofol induces only transient neuronal apoptosis and deficits with no effects on long‐term cognitive impairment. However, repeated propofol exposure can not only induce severe suppression of neurogenesis and apoptosis but also lead to long‐term cognitive impairment. These results suggest that persistent neuronal deficits and disturbances in synapse formation but not transient neuronal apoptosis may contribute to long‐term cognitive impairment. Yang et al. (2014) also demonstrated that propofol induced significant apoptosis in the developing brain but had no effect on cognitive function. Moreover, Shao et al. (2014) also found that propofol might improve cognitive function by attenuating Aβ‐induced mitochondria dysfunction in both aged WT and AD Tg mice. Furthermore, this study also found that AMN‐107 enhanced the number of platform crossings without affecting latency. Nilotinib (AMN‐107, Tasigna), a second‐generation c‐Abl tyrosine kinase inhibitor, was approved by the FDA in 2007 (Weisberg et al., 2019). The effect of AMN‐107 may be closely connected with its ability to improve motor and cognitive symptoms, attenuate neuroinflammation, and reduce the accumulation of neurotoxic proteins in animal models of Parkinson's disease (PD) and AD (Hebron, Lonskaya, & Moussa, 2013; Lonskaya, Hebron, Desforges, Schachter, & Moussa, 2014; Lonskaya, Hebron, Selby, Turner, & Moussa, 2015).

### 4.1 Limitations

Our research has several limitations. First, our study examined only the effects of ROS on the expression of c‐Abl, and the effects of other mechanisms, such as neuroinflammation, on c‐Abl expression require further study. Second, we did not address any sex‐related differences as all of the animals were male. Finally, our study examined the effects of propofol only on cognitive function by propofol‐mediated regulation of c‐Abl expression in a nonsurgical experimental model. Further study will be required to clarify the effects of other anesthetics on the expression of c‐Abl, and models will be needed to address the above questions.

In conclusion, repeated propofol anesthesia did not affect memory D learning function in infant rats by its inhibition of c‐Abl expression. Mechanistically, this effect is mainly related to the antioxidant effect of propofol. Our findings may provide new ideas and methods to prevent postoperative cognitive dysfunction and reductions in learning and memory function after anesthesia.

## CONFLICT OF INTEREST

The authors declare that they have no competing interests.

## AUTHOR CONTRIBUTIONS

Hong Zhang and Longhe‐Xu contributed to the design of the study and the review of the manuscripts. Long Feng and Zhi‐gao Sun mainly completed all experimental operation parts, data analysis, and writing of the first draft of the paper. Qiang‐wei Liu and Tao Ma conducted the animal behavioral experiment. Zhi‐peng Xu, Ze‐guo Feng, and Wei‐xiu Yuan provided good guidance and suggestions for the completion of the experiment. Long Feng and Zhi‐gao Sun contributed equally to this work.

### Peer Review

The peer review history for this article is available at https://publons.com/publon/10.1002/brb3.1810.

## Data Availability

The data used during the study are available from the corresponding author by reasonable request.
